# Preclinical study of simultaneous pharmacokinetic and pharmacodynamic herb-drug interactions between Yin-Chen-Hao-Tang and spironolactone

**DOI:** 10.1186/s12906-020-03042-y

**Published:** 2020-08-15

**Authors:** Tun-Pin Hsueh, Tung-Hu Tsai

**Affiliations:** 1 Institute of Traditional Medicine, School of Medicine, National Yang-Ming University, 155, Linong Street, Sec. 2, Peitou, Taipei, 11221 Taiwan; 2 Department of Chinese Medicine, Kaohsiung Chang Gung Memorial Hospital and Chang Gung University College of Medicine, 123, Dapi Rd. Niaosong Dist, Kaohsiung, Taiwan; 3 Graduate Institute of Acupuncture Science, China Medical University, Taichung, 40402 Taiwan; 4 School of Pharmacy, Kaohsiung Medical University, Kaohsiung, 807 Taiwan; 5 Department of Chemical Engineering, National United University, 2, Lienda, Miaoli, 36063 Taiwan

**Keywords:** *Artemisia*, Drug interaction, Herbal medicine, Spironolactone, Liver disease

## Abstract

**Background:**

The prevalence and therapeutic effects of the use of herbal remedies for chronic liver diseases make the combined administration of herbal products with conventional treatment unable to be ignored. This study investigated the pharmacokinetic and pharmacodynamic herb-drug interactions between the herbal formula Yin-Chen-Hao-Tang (YCHT) and spironolactone.

**Methods:**

A selective high-performance liquid chromatography (HPLC) method was developed and validated for the detection of spironolactone and its metabolite canrenone in rat urine. The interaction study was conducted by collecting urine samples after oral administration of spironolactone alone or in combination with YCHT for 5 days. Urine pharmacokinetic parameters and urinary sodium, potassium, volume, and weight were analyzed.

**Results:**

The results revealed significant increases in the cumulative amount and the area under the rate curve (AURC) of the metabolite canrenone after pretreatment with the high dose of YCHT. The urine weight and volume were significantly reduced dose-dependently as a result of pretreatment with YCHT. The urinary sodium-to-potassium ratio, which indicates diuretic effects, was also reduced in the high-dose YCHT condition.

**Conclusions:**

Herb-drug pharmacokinetic and pharmacodynamic interactions between YCHT and spironolactone were observed in the study. The herb-drug interaction that appeared with a single dose of spironolactone should be considered when patients are being treated with a continuous administration of this drug.

## Background

Liver cirrhosis is the consequence and the end stage of chronic liver disease. The main causes of chronic liver disease in Europe are viral hepatitis B and C, excessive alcohol consumption, and metabolic syndrome combined with obesity [1]. The severity of liver cirrhosis is attributed to the silent course of repetitive pathological deconstruction and regeneration of liver parenchyma until decompensation occurs. The decompensation of cirrhosis, defined as the onset of ascites, portal hypertensive bleeding or encephalopathy, indicates a worsening clinical course and a lower survival rate, especially during follow-up compared to decompensation of cirrhosis at diagnosis [2]. The first and most common complication of decompensation is ascites, both at diagnosis and during follow-up [2]. Ascites is associated with not only poor quality of life but also increased risk of infection, renal failure and poor long-term outcomes [3, 4]. General management of ascites includes sodium intake reduction or low doses of diuretics [5–7]. Although paracentesis is faster, is associated with fewer adverse events and has been shown to be more effective for large volume ascites in randomized trials, diuretics should be administered as maintenance therapy to prevent ascites recurrence, and no difference was observed in long-term mortality between the two strategies [8, 9]. The recommended diuretics are spironolactone (50–200 mg per day) or amiloride (5–10 mg per day) for moderate-volume ascites, and higher doses of 400 mg of spironolactone and 160 mg of furosemide per day are recommended for large-volume ascites [10]. Controlled studies found that spironolactone results in better natriuresis than frusemide [11]. Therefore, spironolactone is the first-line treatment for ascites due to cirrhosis unless it fails to result in a response [12].

Approximately 5.5 million Americans were living with cirrhosis or chronic liver disease (CLD) in 2004 [13]. Individuals with chronic liver disease seek complementary and alternative medicine (CAM) treatments due to the limited efficacy of conventional therapy. A population-based surveillance study enrolled people newly diagnosed with CLD from 1999 to 2001 and found that 16.8% of patients used herbal medicines as a form of CAM therapy. The etiology of CLD in those using CAM was mainly attributed to hepatitis B (33.3%), alcohol use (32.9%), and hepatitis C (31.0%) [14]. A cross-sectional study of the National Health Insurance Research Database in Taiwan revealed that the common single-herb product *Artemisia capillaris* (Yin-Chen-Hao) is a frequently prescribed medicinal herb for diseases ranging from hepatitis B and hepatitis C to hepatocellular carcinoma (HCC) [15–17]. This herbal medicine has been reported to not only ameliorate HBV activity but also improve dyslipidemia in rats with high-fat-diet-induced obesity [18, 19]. Studies have found that the therapeutic properties of *A. capillaris* are attributed to its active compounds, including scoparone (6,7-dimethylesculetin), capillarin, capillarisin, β-sitosterol, quercetin, and cirsimaritin [20]. One of the most important bioactive compounds, scoparone (6,7-dimethylesculetin), has shown anti-inflammatory activities, antioxidant properties, and hepatoprotective properties against hepatitis [21–23]. *A. capillaris* Thunb accompanied by *Gardenia jasminoides* Ellis (Zhi-Zi) and *Rheum officinale* Baill (Da-Huang), referred to as Yin-Chen-Hao-Tang (YCHT, an *A. capillaris* decoction), is a basic herbal formula that is a common prescription in the clinic. The formula was used for hepatitis and jaundice thousand years ago and preserves the established therapeutic properties of *A. capillaris*. Several studies have shown that this formula has protective effects against chlorpromazine-induced cholesteric liver injury [24], the ability to alleviate hepatic oxidative stress [25], and antifibrotic effects in bile duct ligation or dimethylnitrosamine (DMN)-induced liver fibrosis [26, 27]. Adding the other two herbs seems to not only preserve the essential therapeutic properties of *A. capillaris* Thunb but also pharmacokinetically enhance the absorption of scoparone [28].

Since herbal products have been shown to have therapeutic properties and because their use has become more prevalent in the context of chronic liver disease, the safety of combined treatment with herbal products and conventional medicines should not be ignored. Previous articles revealed possible herb-drug interactions of traditional herbal formulas for chronic hepatitis with conventional drugs [29, 30]. For instance, herbal products could delay the elimination of 5-FU in blood or change the bioavailability of carbamazepine [31, 32]. The herbal formula YCHT have been proved to have hepatoprotective and antifibrotic effect. The decision to combine YCHT for its beneficial effects on chronic liver disease with spironolactone to prevent ascites could be argued. Potential interactions occur between the components of herbal products and a single drug that could affect both pharmacokinetics and pharmacodynamics in practice. Given the limited knowledge on the safety of the combined administration of YCHT with spironolactone, the hypothesis of this study lies in a simultaneous investigation of the pharmacokinetic and pharmacodynamic interactions of the herbal product Yin-Chen-Hao-Tang and the diuretic drug spironolactone.

## Methods

### Chemicals and reagents

A reference standard of spironolactone was purchased from Research Biochemicals International. Canrenone (purity ≥97%) and the internal standard n-propylbenzene were obtained from Sigma-Aldrich Inc. All chemicals were of analytical grade, including formic acid (98–100%) and ethanol, which were purchased from E. Merck. The pharmaceutical herbal powder formula Yin-Chen-Hao-Tang was purchased from Kaiser Pharmaceutical Co., Ltd. and was quantified by a Shimadzu UHPLC system coupled to an electrospray ionization (ESI) source equipped with an LCMS-8030 triple quadrupole mass spectrometer to guarantee the consistent quality of the herbal formula powder [33].

### HPLC instrumentation and method validation

The high-performance liquid chromatography (HPLC) equipment for urine analysis consisted of a CBM-20A system controller, LC-20 AD XR pumps, a DGU-20A3 degasser, a SIL-20 AC XR autosampler, and a CTO-20A column oven coupled with a UV detector SPD-M20A. The UHPLC conditions included a reverse-phase C18 column (Purospher STAR, 100 mm × 2.1 mm, 2 μm, Merck, Darmstadt, Germany) and a mobile phase of 0.1% formic acid–MeOH (43:57, v/v) set at a flow rate of 0.2 mL/min for analyte separation. The detection wavelength was set at 254 nm, and the total run time was 15 min. The method validation of the bioanalytical assays for pharmacokinetic studies was performed according to the bioanalysis guidelines from the US FDA [34].

### Animals and experimental design

The adult male Sprague-Dawley rats used in this study were obtained from the Laboratory Animal Center at National Yang-Ming University (Taipei, Taiwan), and the animal experiments were approved by the Institutional Animal Experimentation Committee of National Yang-Ming University (IACUC 1050503, approval on May third 2016). Rats (6–7 weeks old, 250 ± 50 g) were randomly assigned to four groups (six rats of each group) and housed in cages with free access to food and water. In the vehicle group, the rats received no treatment with the same volume of distilled water for 5 days and no spironolactone on the 5th day. The control group was the same but administered 20 mg/kg spironolactone by gavage on the 5th day. Rats in the YCHT group were orally administered 1 g/kg or 3 g/kg of YCHT per day, which corresponded with the intake of 9.7 g or 29.2 g a day, respectively, in a 60-kg human. After YCHT was administered for 5 days, spironolactone was also administered to the rats in these two groups. Urine samples were collected 0–4, 4–8, 8–12, 12–16, 16–20, 20–24, and 24–32 h after the rats in the three groups received 20 mg/kg spironolactone. Samples at each interval were prepared for subsequent analysis. At the end of the experiment, rats were euthanized using carbon dioxide.

### Sample preparation

Urine concentrations of spironolactone and its metabolite canrenone were determined by using a liquid-liquid extraction procedure followed by high-performance liquid chromatography-ultraviolet (HPLC- UV) analysis. First, a 50 μL aliquot of each urine sample was spiked with 10 μL of the IS and added to 250 μL of ethyl acetate. Then, the mixture was vortexed for 5 min and centrifuged at 13000 rpm for 10 min at 4 °C. The procedure was repeated twice for effective phase separation. The supernatant was transferred to a rotary evaporator for an hour at 50 °C. Following the procedure, the residues were reconstituted in 100 μL of 50% methanol and injected into the HPLC system for analysis.

### Pharmacodynamics analysis

To simultaneously acquire the pharmacokinetic and pharmacodynamic data, urine samples from each interval following dose administration were obtained. The response to the diuretic was monitored and included urine weight, urine volume, and the concentration of sodium and potassium at each collection interval. The level of urinary sodium and potassium were determined using an automated urinalysis analyzer in a clinical laboratory. Patients with ascites usually require 24-h urine collection to evaluate urinary sodium secretion. The sodium-to-potassium ratio was considered a replacement for the collection of 24-h urine samples because the correlation between 24-h sodium and urinary sodium-to-potassium ratio concentration was significant and showed 75% sensitivity and 91.67% specificity [35]. The urinary sodium-to-potassium ratio is also a translatable quantitative biomarker of mineralocorticoid receptor antagonism and is used to determine the potential value of spironolactone [36]. The sodium and potassium levels in urine that correspond to the effects of spironolactone were assessed by the ratio of urinary sodium-to-potassium concentration.

### Pharmacokinetic analysis

Pharmacokinetic parameters were calculated using WinNonlin version 1.1 (Scientific Consulting Inc., Apex, NC, USA) with a noncompartmental model. The urine volume and concentration of urine at each collection interval were measured directly from the experimental data. The cumulative amount of spironolactone and canrenone excreted was calculated as the observed concentration times the urine volume of each collection period and was plotted against the median of the collection interval. The total cumulative amount observed (Cum) and its estimated percentage versus administration dose (Cum %) were also calculated. The urinary excretion rates were determined at 4-h intervals over the entire dosing interval. The maximum observed excretion rate (R_max_) and the midpoint of the collection interval associated with R_max_ (T_max_) were acquired from the excretion rate data. Pharmacokinetic parameters estimated from the profiles of the individual urinary excretion rate over time included the area under the urinary excretion rate curve from time zero to the last sampling time (AURC_0–32_) and from time zero to infinite time (AURC _0-∞_). The elimination rate constant (K_e_) was assessed by log-linear regression of the terminal portion of the excretion rate versus time curve, and the elimination half-life (t_1/2_) was the product of ln2 divided by K_e_.

### Statistical analysis

Data in the study are presented as the mean ± standard deviation (SD). Comparisons between two groups were performed by using the unpaired two-tailed *t*-test. Analysis of variance (ANOVA) was employed to compare more than two groups to analyze the difference among group means. Differences were considered to be statistically significant for *p*-values lower than 0.05 (*p* < 0.05).

## Results

**Fig. 1:**
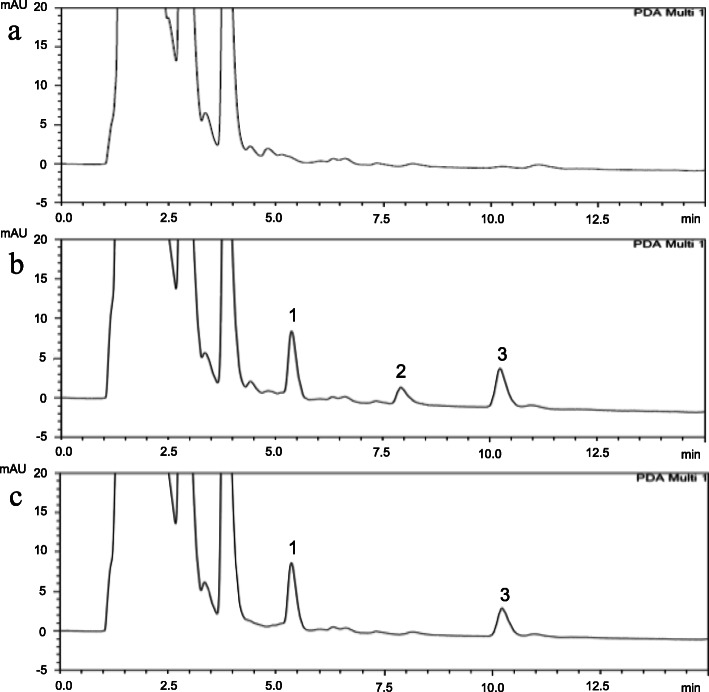
HPLC chromatograms of (**a**) drug-free urine extract; (**b**) urine sample spiked with spironolactone (10 μg/mL; RT: 8.56 min), canrenone (10 μg/mL; RT: 11.11 min) and n-propylbenzene (10 μg/mL; IS, RT: 5.61 min); and (**c**) urine extract after the administration of spironolactone for 12–16 h (20 mg/kg, p.o.). 1: n-propylbenzene; 2: spironolactone; 3: canrenone

The chromatographic separation achieved good linearity (R^2^ > 0.999) in the range of 0.25 μg/mL to 50 μg/mL. The limits of detection (LODs) of spironolactone and canrenone were 0.1 and 0.25 μg/mL, respectively. The retention times of spironolactone, canrenone and internal standard n-propylbenzene were 8.56 min, 11.11 min, and 5.61 min, respectively, and no interference existed in the analytical conditions (Fig. 1). The precision and accuracy guaranteed the reproducibility of the developed analytical method. For spironolactone and canrenone, the intraday assay results for precision ranged from 0.10–7.88 and 0.05% - 10.76%, respectively, while those for accuracy ranged from − 1.03 – 3.72% and − 7.28 – 4.07%, respectively. The interday assay results for spironolactone and canrenone were 0.23–10.42% and 1.48–11.29%, respectively, for precision and − 1.10 – 4.99% and − 5.21 – 2.05%, respectively, for accuracy. The recovery of the spironolactone and canrenone concentrations in rat urine was consistent independent of low or high concentrations. The results of precision and accuracy were all within ±15% of nominal values that showed good reproducibility for quantification with this analytical method.

**Fig. 2:**
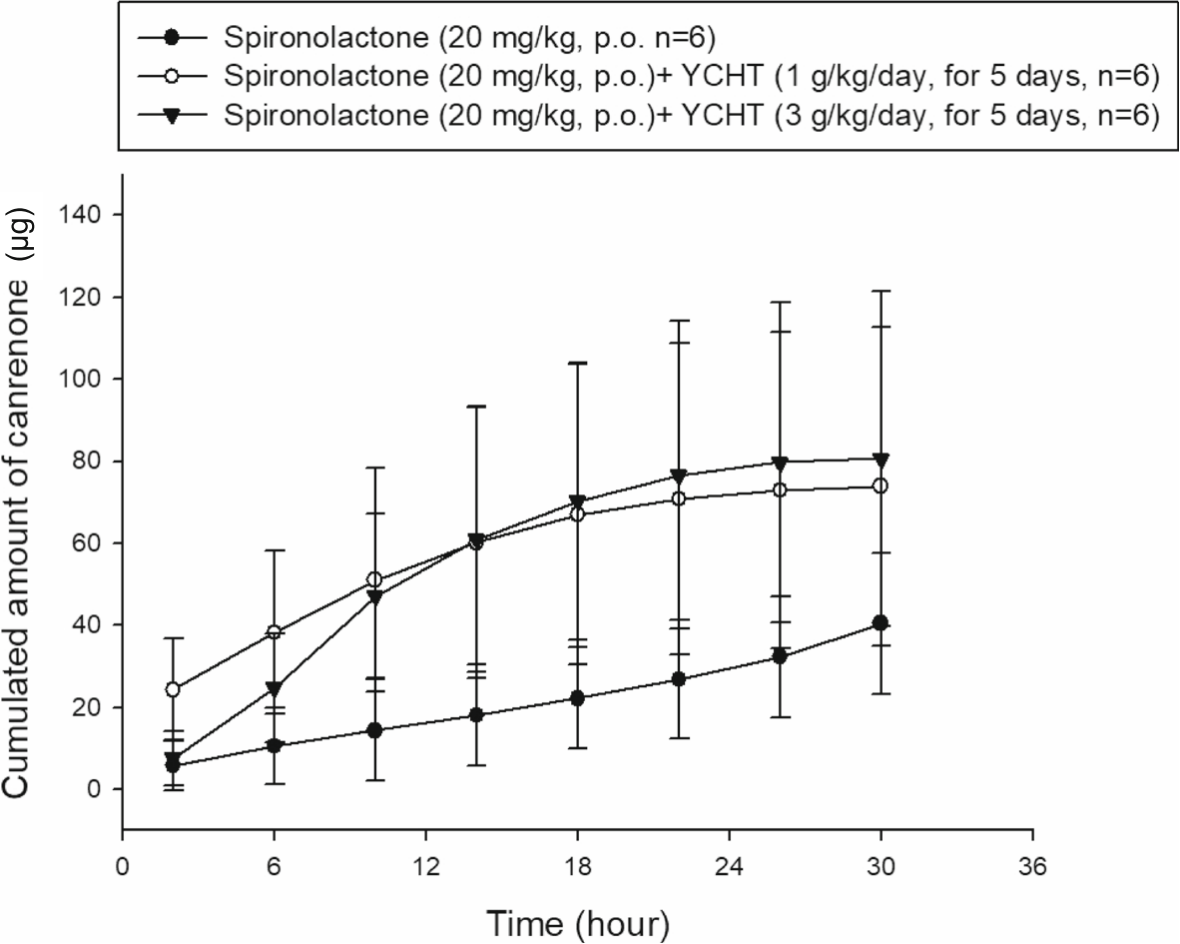
The cumulative amount of canrenone excreted in rat urine after administration of spironolactone. The figure illustrates the cumulative urinary amount of canrenone versus the time midpoint after administration of spironolactone in the control and YCHT pretreatment groups

The newly developed HPLC method was applied in the following pharmacokinetic study of rat urine. In our experiment, only the metabolite canrenone was found in rat urine. The pharmacokinetic parameters revealed that the observed cumulative amount of canrenone increased as the dose of YCHT increased. The mean cumulative amount of canrenone in the control group was 40.4 ± 17.2 μg, which accounted for approximately 0.2% of the dose. The cumulative amount of canrenone in the urine was 74.2 ± 39.3 μg with pretreatment with 1 g/kg YCHT and showed no difference from the control group. However, the cumulative amount in the 3 g/kg YCHT group reached 80.1 μg, which was significantly higher than that in the control group (Fig. 2). The area under the rate curve (AURC) also revealed a significant difference resulting from high dosing of YCHT. The mean observed AURC was 67.5 ± 36.5 μg in the 1 g/kg YCHT group and 34.9 ± 14.8 μg in the control group. The AURC of the 3 g/kg YCHT group reached 78.1 ± 38.8 μg, which was similar to the results of the urinary cumulative amount. In addition, the excretion rate of canrenone as well as the maximum rate and the terminal elimination half-life did not change significantly after intervention with the different doses of YCHT (Table 1).

**Table 1:** Pharmacokinetic parameters of canrenone in rat urine after single oral administration of spironolactone

Parameters	Spironolactone	YCHT 1 g/kg + Spironolactone	YCHT 3 g/kg + Spironolactone
Cum (μg)	40.4 ± 17.2	74.2 ± 39.3	80.1 ± 39.5 *
Cum% (% kg)	0.20 ± 0.09	0.37 ± 0.20	0.40 ± 0.20 *
R_max_ (μg/hr)	3.67 ± 2.07	6.34 ± 3.00	6.02 ± 2.08
T_max_ (hr)	15.3 ± 12.0	3.33 ± 3.27 *	10.0 ± 4.38
AURC _0–32_ (μg)	34.9 ± 14.8	67.5 ± 36.5	78.1 ± 38.8 *
R_avg_ (μg/hr)	1.26 ± 0.36	2.31 ± 1.93	2.52 ± 1.82
K_e_ (hr^− 1^)	0.09 ± 0.06	0.19 ± 0.14	0.36 ± 0.30
t _1/2_ (hr)	12.6 ± 12.0	5.05 ± 2.87	3.77 ± 2.96

Data are expressed as the mean ± S.D. (*n* = 6)

Data were significantly different by t-test. * *p* value < 0.05

*Cum* Cumulative amount of canrenone excreted within 32 h., *Cum%* Percent of cumulative amount excreted versus administration dose, *R*_*avg*_ Average rate of excretion, *R*_*max*_ Maximum rate of excretion, *T*_*max*_ Time of maximum rate of excretion, *AURC*_*0–32*_ Area under the rate of excretion versus midpoint of time interval curve for time 0 to 32 h, *K*_*el*_ Elimination rate constant, *t*_*1/2*_ Elimination half-life, *YCHT* Chinese herbal formula Yin-Chen-Hao-Tang

**Fig. 3:**
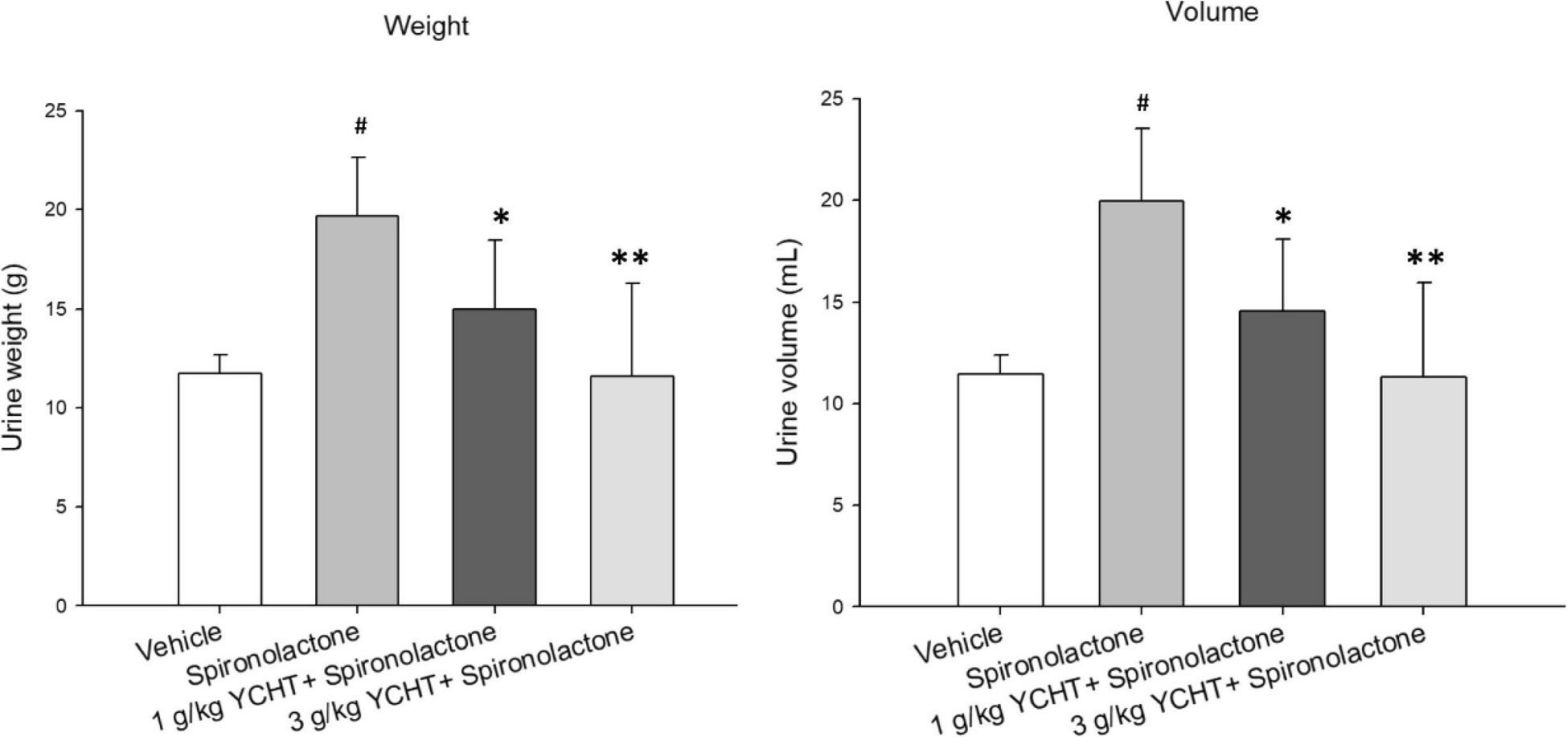
Total urine weight and volume after oral administration of spironolactone in each group. Data were significantly different by *t*-test. # indicates a *p* value less than 0.01 compared with the vehicle group; * indicates a p value less than 0.05 and ** indicates a p value less than 0.01 compared with the spironolactone group. YCHT: Chinese herbal formula Yin-Chen-Hao-Tang

The total urine volume and weight were significantly increased by spironolactone treatment in the control group. The cumulative urine excretion at 32 h decreased by 24% in weight and 27% in volume in the pretreatment with 1 g/kg YCHT for 5 days group compared to that in the control group and decreased by 41 and 43% in weight and volume, respectively, in the 3 g/kg YCHT group (Fig. 3). Generally, the urine weight and urine volume were significantly impacted by the increasing dose of YCHT.

**Fig. 4:**
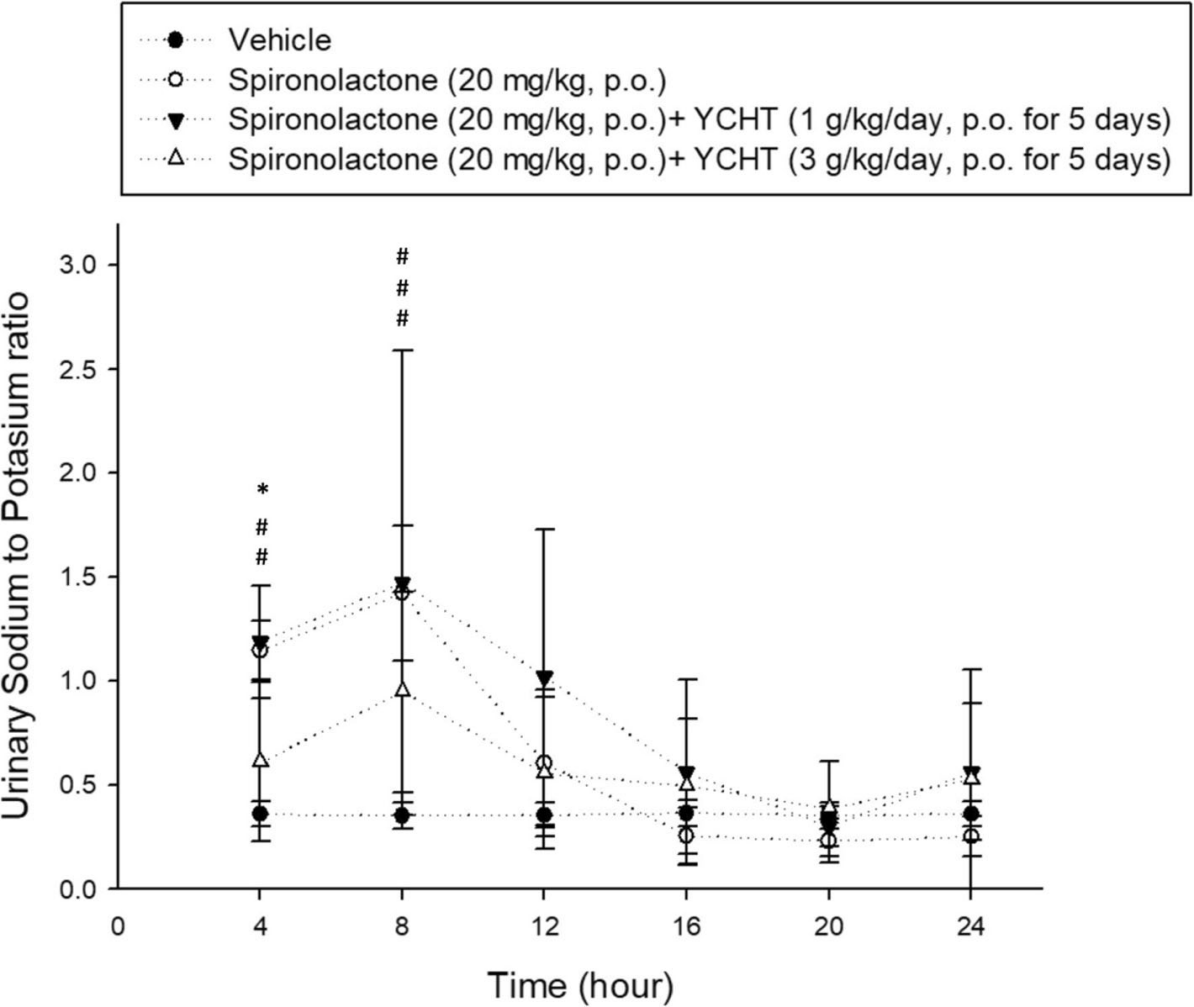
Urine sodium-to-potassium ratio of each group over 24 h. Each symbol represents an experimental group. Data were significantly different by *t*-test. # indicates significance compared with the vehicle group; * indicates significance compared with the spironolactone group. The sodium-to-potassium ratio of only the 3 g/kg YCHT group was significantly different than that in the spironolactone group. YCHT: Herbal formula Yin-Chen-Hao-Tang

The urinary sodium-to-potassium ratio of each interval over 24 h was observed after a single dose of spironolactone. The urinary sodium-to-potassium ratio was significantly increased by spironolactone in the control group during the first 8 h compared to the ratio in the vehicle group, and it was also significantly increased in the 1 g/kg YCHT group (*p* < 0.0001). However, the urinary sodium-to-potassium ratio did not increase in the pretreatment with 3 g/kg YCHT group during the first 4 h (*p* < 0.01) and remained nonsignificantly different from the vehicle. The urinary sodium-to-potassium ratio over 4 to 8 h in the 3 g/kg YCHT group was then significantly inversely associated with that in the vehicle group, as it was in the control group. The urinary sodium-to-potassium ratio after 8 h did not show any significant difference among the four groups (Fig. 4). The pharmacodynamics in the urinary sodium-to-potassium ratio seemed to correspond with the pharmacokinetics results.

## Discussion

The newly developed HPLC method was validated and achieved good linearity with reliable accuracy and precision; thus, it was sufficient for use in further pharmacokinetic analysis without compromised pharmacodynamic acquisitions. The experimental results showed that the pharmacokinetic parameters of the cumulative amount and AURC of canrenone were significantly increased by pretreatment with high-dose YCHT. Additionally, YCHT interfered with the diuretic effect of spironolactone, and the urinary sodium-to-potassium ratio was also compromised by the high-dose YCHT treatment. The simultaneous pharmacokinetic and pharmacodynamic herb-drug interactions between YCHT and spironolactone were demonstrated by the study.

The pharmacokinetic parameters revealed that the cumulative amount and AURC of canrenone were significantly increased in urine but that the excretion rate and the mean terminal elimination half-life were not significantly affected by the high dose of YCHT. These results indicated that the high dose of YCHT affected only the amount of canrenone inside the body without altering the excretion capacity, which was still far from saturation under the YCHT pretreatment condition. The changes in excretion amount with a maintained excretion rate suggested that the herb-drug interaction probably did not occur at the renal level. A partial explanation of this may lie in the previously reported study that revealed that the sulfoxidation reactions of spironolactone were diminished in biliary fistula patients and that the dethioacetylation reaction yielding canrenone in urine was consequently enhanced [37]. This previous study proposed that biliary drainage interrupted the enterohepatic circulation and led to a significant reduction in all sulfoxidation reactions, increasing the common precursor 6β-OH-7α-thiomethyl spironolactone on the one hand and enhancing dethioacetylation yielding canrenone on the other hand. Scoparone has cholagogue properties, and its highest levels were found in the liver; scoparone is a main bioactive component of YCHT [24, 38]. Our results of the high-dose YCHT group were similar to the findings of this previous study showing that the excretion of canrenone was significantly increased. Consequently, the pharmacokinetic interaction of YCHT with spironolactone possibly lies in the alternation of spironolactone metabolism in the liver.

The herb-drug pharmacokinetic interaction subsequently affected urinary electrolyte excretion caused by potassium-sparing spironolactone. Only the metabolite of spironolactone was detected in the urine. The rapid elimination of spironolactone was noticed in the study and was therefore considered to have contributed to the increase in the urinary sodium-to-potassium ratio in the first 8 h. The sodium-to-potassium ratio was significantly increased due to the excessive sodium excretion caused by spironolactone but was reduced in the first 4 h by the 3 g/kg YCHT pretreatment. The result was similar to the pharmacokinetic results, indicating an insufficient dose effect of 1 g/kg YCHT on spironolactone. The explanation would be supported by the previous pharmacokinetic study of YCHT that showed that increasing the dose of this herbal formula would largely increase the maximum concentration and bioavailability of its bioactive constitutions [39]. The interference with the sodium-to-potassium ratio in the 4 to 8 h after administration of spironolactone then diminished, possibly due to the maximum retention time of approximately 2 h of YCHT. The high bioavailability of YCHT not only affected the pharmacokinetics of spironolactone but also the pharmacodynamics of electrolytes.

Furthermore, the urine weight and volume decreased significantly with the dose of YCHT. These results were not the same as the results of pharmacokinetics and those showing that urinary sodium and potassium concentrations are closely related to spironolactone. This phenomenon may imply an anti-diuretic effect of YCHT that requires further basic research. The herb-drug interaction discovered in the animal study that only provided a basis for clinical studies. A closely monitoring prospective clinical trial should be needed for the adverse effects of coadministrations of this drug.

## Conclusions

Pharmacokinetic and pharmacodynamic experiments through HPLC analysis illustrated an herb-drug interaction between YCHT and spironolactone. The urinary cumulative amounts of the metabolite canrenone increased under a high dose of YCHT treatment. High-dose pretreatment with YCHT also interfered with the urinary sodium-to-potassium ratio of spironolactone. The herb-drug interaction that appeared at a single dose of spironolactone thus should indicate the need for caution while patients are under the continuous administration of this drug. The desire to improve therapeutic effects and care in dose management of YCHT should be balanced when using these herbs together with spironolactone.

## Data Availability

All data generated or analyzed during this study are included in this published article and its supplementary information files.

## Abbreviations

AURC: The area under the rate curve
CLD: Chronic liver disease
CAM: Complementary and alternative medicine
Cum: Cumulative amount
HCC: Hepatocellular carcinoma
K_e_: The elimination rate constant
LOD: The limit of detection
R_max_: The maximum observed excretion rate
T_max_: The midpoint of the collection interval associated with R_max_
R_avg_: The average excretion rate
YCHT: Yin-Chen-Hao-Tang
